# The double PHD finger domain of MOZ/MYST3 induces α-helical structure of the histone H3 tail to facilitate acetylation and methylation sampling and modification

**DOI:** 10.1093/nar/gkt931

**Published:** 2013-10-21

**Authors:** Ingrid Dreveny, Sian E. Deeves, Joel Fulton, Baigong Yue, Marie Messmer, Amit Bhattacharya, Hilary M. Collins, David M. Heery

**Affiliations:** Centre for Biomolecular Sciences, School of Pharmacy, University of Nottingham, Nottingham NG7 2RD, UK

## Abstract

Histone tail modifications control many nuclear processes by dictating the dynamic exchange of regulatory proteins on chromatin. Here we report novel insights into histone H3 tail structure in complex with the double PHD finger (DPF) of the lysine acetyltransferase MOZ/MYST3/KAT6A. In addition to sampling H3 and H4 modification status, we show that the DPF cooperates with the MYST domain to promote H3K9 and H3K14 acetylation, although not if H3K4 is trimethylated. Four crystal structures of an extended DPF alone and in complex with unmodified or acetylated forms of the H3 tail reveal the molecular basis of crosstalk between H3K4me3 and H3K14ac. We show for the first time that MOZ DPF induces α-helical conformation of H3K4-T11, revealing a unique mode of H3 recognition. The helical structure facilitates sampling of H3K4 methylation status, and proffers H3K9 and other residues for modification. Additionally, we show that a conserved double glycine hinge flanking the H3 tail helix is required for a conformational change enabling docking of H3K14ac with the DPF. In summary, our data provide the first observations of extensive helical structure in a histone tail, revealing the inherent ability of the H3 tail to adopt alternate conformations in complex with chromatin regulators.

## INTRODUCTION

Histone N-terminal tails are subject to multiple posttranslational modifications (PTMs) that can modify chromatin structure and act as signals to recruit, evict or repel chromatin regulators. Thus, histone PTMs constitute a combinatorial ‘semaphore’ that demarcates genomic regions for activation, repression, repair or other processes (1). Chromatin regulatory complexes can establish or erase these signals through their enzymatic activities, and many contain reader domains that enable them to recognize selected histone PTMs (2). Dynamic changes in histone PTMs reflect the expression status of genes and their regulatory regions, thus the ability of chromatin regulators to recognize combinatorial heterotypic PTMs in their histone substrates is essential to their function. Supporting evidence for this model comes from structural studies of histone tail recognition by the tandem bromodomains of bromodomain, testis specific (BRDT) (3), the plant homeodomain (PHD finger) and double tudor domains of ubiquitin-like with PHD finger and RING domains 1 (UHRF1) (4) and the dual PHD/Bromo domains of bromodomain PHD finger transcription factor (BPTF) (5). A systematic study of the interactions of bromodomains with acetylated histone peptides suggests that multivalent recognition of PTMs is an important and widespread function of chromatin readers (6). This sampling functionality is likely to underpin crosstalk between different histone PTM signals, facilitating recruitment and dismissal of chromatin regulators to drive genomic processes. Understanding these processes at the molecular level will be essential for devising new therapeutic interventions in human diseases such as cancer, in which chromatin functions are disrupted.

The developmental regulator monocytic leukaemia zinc finger protein (MOZ) also known as (MYST3/KAT6A) is a MYST (Moz, Ybf, Sas, TIP60) family acetyltransferase that is required for self-renewal and differentiation of haematopoietic stem cells (7). MOZ appears to function as a cofactor for AML1, Pu.1 and p53-mediated gene expression (8–10). Recurrent translocations within the MOZ gene are associated with acute leukaemia, producing oncogenic MOZ fusion proteins that can induce leukaemia in animal models (11,12). Fusion proteins such as transcriptional intermediary factor 2 (MOZ-TIF2) retain the N-terminal portion of MOZ and show aberrant functionality, which impacts on histone modification and gene regulation (8,10,13).

**Figure 1.**
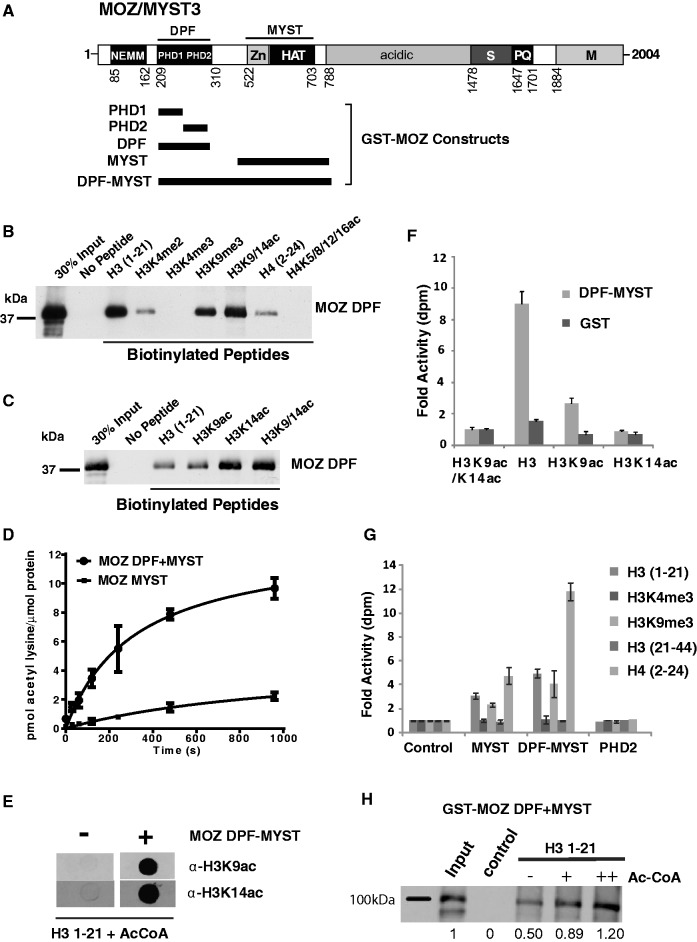
MYST DPFs function in histone acetylation and PTM recognition. (**A**) MOZ/MYST3 domain structure showing the NEMM (N-terminal domain of Enoki, MOZ and MORF); DPF; and MYST domains, the latter of which combines a zinc finger (Zn) and HAT domain. Regions rich in serine (S), proline and glutamine (P/Q), methionine (M) or acidic residues are also indicated. Approximate boundaries of GST-fusion proteins used for *in vitro* assays are also indicated. (**B**) MOZ DPF domains sense H3 and H4 PTMs. *In vitro* binding assays showing the interaction of purified GST fusion proteins with immobilized biotinylated H3 and H4 peptides, either unmodified or bearing specific modifications, as indicated. Affinity capture of recombinant GST-MOZ DPF proteins on histone peptides is detected by western blotting using α-GST antibody. (**C**) Effect of H3 acetylation on binding of GST MOZ DPF (**D**) MOZ DPF enhances histone acetylation by the MYST domain. *In vitro* HAT assays showing rates of acetylation of core histones by equimolar amounts of MOZ MYST domain (510–810) or the combined DPF-MYST (194–810). The data represent the mean of replicates and error bars show standard deviations. (**E**) MOZ DPF-MYST acetylates H3K14 and H3K9. Immunodetection of H3K9ac and H3K14ac after treatment of unmodified H3 with GST-MOZ-DPF, or GST control. (**F**) *In vitro* HAT assays showing acetylation of unmodified H3, H3K9ac and H3K14ac peptides by MOZ DPF-MYST domain, or control. (**G**) Acetylation of H3 and H4 peptides by MOZ PHD2, MOZ MYST or MOZ DPF-MYST proteins or control. Data columns appear in order listed in the key. (**H**) H3 tail acetylation enhances binding by MOZ DPF-MYST. Binding assays of GST MOZ DPF-MYST to unmodified H3 in the presence 0, 1.5 or 3 µM acetyl CoA. Proportion of affinity captured DPF-MYST proteins relative to input was quantified by densitometry.

In addition to the MOZ acetyltransferase domain (referred to as the MYST domain), the N-terminus of MOZ contains two tandem PHD fingers comprising the double PHD finger (DPF) domain (Figure 1A). While PHD2 shares homology with numerous other PHD fingers, PHD1 is distinctive, showing closest homology with MOZ related factor (MORF)/MYST4 and the BRG1 associated factor (BAF) complex components DPF1/BAF45b/neuro-d4, DPF2/BAF45d/ubi-d4, DPF3/BAF45c/cer-d4 and PHF10/BAF45a, all of which contain a similar tandem PHD finger arrangement (for sequence alignment, see Figure 5D). The DPF3b isoform was shown to have acetyl histone-binding function (14), and a subsequent nuclear magnetic resonance structure revealed how its PHD fingers function together to achieve combinatorial recognition of unmodified H3K4 and acetylated H3K14 (15). The H3 tail in these solution structures adopted an extended conformation as has been observed for H3 and H4 histone tail peptides in complex with other PHD domains (16) (see Figure 5B and E). MOZ also binds acetylated histone H3 tails (17,18) and a crystal structure in complex with an H3 peptide-reported DPF interactions with the first five residues of H3 (18). However, how MOZ engages the H3 tail to interpret its methylation/acetylation status remains poorly understood.

In this study, we report a series of four crystal structures of an extended MOZ DPF domain alone and in complex with the H3 N-terminal tail, including unmodified, H3K9ac and H3K14ac forms providing detailed novel insights into the consequences of PTM on H3 tail structure in complex with a DPF. The MOZ DPF domain is expanded in comparison with other studies to include a sequence at the N-terminal boundary that is highly conserved in MORF/MYST4/KAT6B. We show that the H3 N-terminus can adopt significant α-helical conformation in complex with a chromatin regulator, which has not been observed previously. The structures combined with biochemical studies reveal how MOZ DPF manipulates the structure of the H3 tail to sample its modification status and promote its acetylation. The structures also show that docking of H3K14ac with the DPF depends on a double glycine hinge (G12–G13) in H3 that can undergo a conformational change. These results provide new insights into histone tail recognition and acetylation by MOZ, crosstalk between heterotypic histone modifications and reveal novel structural features of the H3 N-terminus and its inherent conformational adaptability in complex with chromatin regulators.

## MATERIALS AND METHODS

### Expression constructs, peptides, antibodies

Glutathione-S-transferase (GST)-fusion constructs MOZ (1–321); MOZ DPF (194–323); MOZ PHD1 (194–265); MOZ PHD2 (250–323); MOZ MYST (510–810); MOZ DPF-MYST (194–810) and MORF DPF (210–330) were constructed by polymerase chain reaction (PCR) subcloning into the vector pGEX-DMH (19). The expression construct His-MOZ DPF (194–323) was generated by PCR subcloning into the *Xho*I and *Bam*HI sites in pET15b vector (Novagen). FLAG-MOZ full-length (1–2004) and FLAG MOZ-N (1–1117) have been described previously (10). Biotin-conjugated histone peptides were purchased from Millipore and Peptide Protein Research Ltd. Calf thymus core histone preparations were purchased from Sigma or Roche. Antibodies α-GST (G7781) and α-FLAG M2 (F3165) were from Sigma; α-MOZ (KAT6A-61327) was from Active Motif; α-H2A (ab18255), α-H2B (ab1790), α-H3 (ab1791), α-H4 (ab10158), α-H3K9ac (ab4441), α-H3K9me3 (ab8898) and α-H3K4me3 (ab8580) were from Abcam; and α-H3K14ac (20) was a gift from Laszlo Tora.

### Protein expression and purification

His-tagged MOZ DPF recombinant protein was expressed in *Escherichia coli* Rosetta 2. Cultures were grown at 37°C, followed by induction with 0.1 mM Isopropyl β-D-1-thiogalactopyranoside (IPTG) for 16 h at 22°C. Cell pellets were resuspended in binding buffer (0.02 M sodium phosphate, 0.5 M NaCl, pH 7.4), sonicated and the soluble protein was harvested by centrifugation. Recombinant protein was purified on a HiTrap chelating column preloaded with NiSO_4_. The column was equilibrated with binding buffer, loaded with the protein sample and washed with binding buffer containing 5 mM imidazole. His-tagged MOZ DPF was then eluted in binding buffer containing 300 mM imidazole and the His-tag removed by incubation with thrombin overnight at 22°C. The untagged MOZ DPF was purified to homogeneity on a Superdex 75 16/60 gel filtration column (GE Healthcare) using 50 mM Tris–Cl, pH 7.6, 150 mM NaCl, 1 mM dithiothreitol (DTT) as running buffer.

Recombinant GST-tagged MOZ was produced in Rosetta cells. Cultures were grown at 37°C and recombinant protein production was induced using 0.3 mM IPTG for 3–16 h at 20, 30 or 37°C (conditions optimized for each construct). Cells were harvested, resuspended in NTN buffer (20 mM Tris–HCl, pH 8.0, 100–500 mM NaCl, 0.5% NP-40), sonicated and the soluble protein harvested by centrifugation. Recombinant proteins were purified on glutathione sepharose beads (GE Healthcare) on a rotating wheel overnight at 4°C. The beads were collected by centrifugation and GST-fusion proteins eluted with glutathione buffer. The samples were finally desalted and buffer exchanged using PD-10 desalting columns (Sephadex TM G-25 medium, GE Healthcare) and concentrated using Vivaspin concentrators (Sartorius).

### Histone binding, acetylation assays, chromatin IP

For histone peptide binding assays 1.5 μg of purified GST-tagged fusion protein was incubated with 1.5 μg of biotin-conjugated histone peptides (Millipore/Peptide Protein Research) or no peptide as control, overnight with rotation at 4°C. Dynabeads® (M-280 Streptavidin-Invitrogen) were pre-washed twice in 1× phosphate buffered saline (PBS) containing 0.1% Tween 20 and 20 μl added to each reaction for 1 h at 4°C. Acetyl CoA (1.5 or 3 µM) was added if required. The beads were harvested using a magnet and washed (3 × 10 min) in binding buffer. Following sodium dodecyl sulphate-polyacrylamide gel electrophoresis (SDS PAGE), bound proteins were visualized by immunodetection with α-GST, and quantified by densitometry using image J software, as required. Similarly, peptide binding assays were performed with normalized amounts of *in vitro* translated ^35^[S] methionine-labelled FLAG-MOZ proteins, with bound protein visualized by autoradiography. Alternatively, core histones (10 μg) were added to GST pull-downs and bound histones detected by immunoblot using specific antibodies.

For acetylation assays, 1.5 µg of purified GST or GST-MOZ proteins was mixed with 10 µg of core histones or 0.75 μg of histone peptide in 40 µl of histone acetyltransferase (HAT) buffer containing 5 µg of bovine serum albumin. Samples were incubated for 2 min at 30°C before the addition of 1.8 μl of radiolabelled acetyl-CoA [^3^H] (15.4 μM). Reactions were stopped after 30 min by adjusting to 1× SDS PAGE loading buffer and boiling for 2 min. Control reactions to ascertain background included histones only, and GST-MOZ proteins without histones. Incorporation of acetyl[^3^H] in histones or histone peptides was measured in triplicate by scintillation counting. Activity was expressed as the fold increase in decays per minute over control. For kinetic assays, reactions were stopped at the indicated times and expressed as pmoles acetyl[^3^H] incorporated/pmole GST-MOZ protein. To detect specific acetylation events (H3K9ac; H3K14ac) by western blots, HAT assays were performed as above but with unlabelled acetyl-CoA and spotted onto nitrocellulose filters before immunoblotting with specific antibodies.

For chromatin IP, K562 cells were grown in RPMI supplemented with foetal bovine serum. Approximately 1.5 × 10^7^ cells were fixed with 1% formaldehyde for 8 min at room temperature and subsequently quenched with 0.125 M glycine. Cells were washed three times with ice-cold PBS, resuspended in cell lysis buffer [5 mM Tris–Cl (pH 8.0), 85 mM KCl, 0.5% NP-40] and incubated for 10 min on ice. Chromatin was extracted from nuclear pellets using a chromatin immunoprecipitation (ChIP)-IT kit (Active Motif). The sample was sonicated using a Diagenode water bath sonicator under conditions optimized to give a DNA fragment length of 200–500 bp. DNA content was measured using a bioanalyser (Agilent) and fragment size verified by gel analysis. Immunoprecipitation was performed with PureProteome magnetic beads (Millipore) as per manufacturer’s protocol. Approximately 10 µg of chromatin was used for each IP, which was incubated with 5 µg of a monoclonal α-MOZ antibody (α-KAT6A) overnight at 4°C. The beads were washed three times for 10 min with 1× PBS-Tween and the immunoprecipitate was eluted by 60 µl SDS PAGE loading buffer (X4). Samples were boiled for 10 min, removed from beads and processed by SDS PAGE. Immunoblots were then performed to detect for enrichment of specific histone PTMs.

### Crystallization, data collection and structure solution

Pure MOZ DPF fractions were concentrated to 9.4 mg/ml and subjected to crystallization trials. An aliquot of the sample was mixed in a 1:1.5 molar ratio with N-terminal histone H3 peptide spanning residues 1–21, incubated on ice for 30 min and set up for co-crystallization trials. Large single crystals grew after 3 days with 100 mM Na-Hepes, pH 7.5, 1.4 M sodium citrate as precipitant solution. Single crystals were flash cooled in liquid nitrogen using ethylene glycol as cryoprotectant. Crystals have the symmetry of space group P4_3_2_1_2 with cell parameters of a = b = 70.52 Å, c = 96.83 Å. A high resolution data set was collected at the European Synchrotron Radiation Facility (ESRF), France, beam line ID23-2 at a wavelength of 0.8726 Å and at cryogenic temperatures. Data collection and refinement statistics are summarized in Table 1. The structure was solved by single-wavelength anomalous dispersion (SAD) using the anomalous signal from the zinc atoms. Crystals of MOZ DPF in complex with H3K9ac were obtained under equivalent conditions as the unmodified H3 complex crystals. Data to 1.6 Å resolution were collected at the ESRF, beamline ID-29. Apo crystals of MOZ DPF grew in 1:1 ratio of protein solution to mother liquor comprising 60% tacsimate, pH 7 (Hampton Research, USA), at room temperature. Crystals were transferred to a solution of 80% tacsimate supplemented with 10% glycerol before flash cooling. A data set to 3 Å resolution with unit cell parameters of a = b = 64.69 Å, c = 64.80 Å and space group P 3_1_ 2 1 was collected at the ESRF, France, beam line ID23-2 at a wavelength of 0.8726 Å. Crystals of MOZ DPF in complex with H3 K14ac grew in 0.2 M Li_2_SO_4_, 0.1 M Tris–Cl, pH 8.5, and 30% PEG 4000 after 2 months. Crystals have the symmetry of space group C2, unit cell parameters a = 138.72 Å, b = 32.20 Å, c = 74.87 Å, β = 94.36°. A data set at 100 K at a wavelength of 0.91732 Å was collected at Diamond Light Source (DLS, Oxford, UK) beamline I04-1. The MOZ DPF and DPF–H3 complex structures were solved by molecular replacement with the program PHASER using the model generated from the DPF–H3 complex SAD experiment as a search model (21).

**Table 1.** Data collection and refinement statistics

Data set	MOZ DPF	MOZ DPF–H3 (SAD data set)	MOZ DPF–H3 K9ac	MOZ DPF–H3 K14ac
Data collection				
Space group	P 3_1_ 2 1	P 4_3_ 2_1_ 2	P 4_3_ 2_1_ 2	C 2
Cell parameters				
a, b, c (Å)	64.69 64.69 64. 80	70.52 70.52 96.83	70.46 70.46 96.37	138.72 32.20 74.87
α, β, γ (°)	90 90 120	90 90 90	90 90 90	90 94.36 90
Resolution (Å)	56–3.0 (3.16–3.00)	57.0–1.6 (1.79–1.6)	48.19–1.61 (1.64–1.61)	29.1–2.5 (2.64–2.5)
R_merge_ (%)	7.4 (70)	5.7 (47.3)	7.6 (86.2)	15.7 (32)
I/σI	14.9 (2.7)	16.8 (3.6)	19.6 (2.8)	12.4 (3.8)
Completeness (%)	99.7 (99.3)	99.7 (99.7)	100 (100)	99.6 (98.3)
Redundancy	4.5 (4.6)	4.9 (5.0)	11 (10.5)	9.5 (4.1)
Refinement				
Resolution (Å)	56.0–3.0	49.9–1.6	44.3–1.6	29.1–2.5
Number of reflections	3686	32739	32092	11577
R_work_ (%)/R_free_ (%)	23.9/25.5	16.5/18.6	16.5/18.5	21.9/26.6
Number of atoms	945	1234	1267	2283
Protein	935	1070	1105	2127
Ligand/ion	4	4	4	8
Water	6	160	158	148
Average B-factors (Å^2^)				
Protein	84.1	26.2	35.7	41.4
Ligand/ion	79.0	19.3	25.6	32.5
Water	62.4	33.8	44.1	45.9
RMSD				
Bond lengths (Å)	0.004	0.017	0.008	0.005
Bond angles (°)	1.09	1.58	1.26	0.903
Ramachandran				
Favoured region (%)	95	98	98	95
Allowed region (%)	5	2	2	5

Values in parentheses correspond to the highest-resolution shell.

### Model building and structure refinement

After density modification, model building of the MOZ DPF–H3 complex structure was straight forward and was performed using a combination of automatic model building (RESOLVE) and manual rebuilding and adjustments in COOT (22). The structure was refined to a final crystallographic Rfactor of 16.5% and Rfree of 18.6% using the PHENIX suite of programs (23). Ninety-eight percent of residues were in the favoured regions of the Ramachandran plot and 2% in the allowed regions. The DPF in complex with H3K9ac was refined to an Rfactor of 16.5% and Rfree of 18.5%, with 98% of residues in the preferred and 2% of residues in the allowed regions of the Ramachandran plot. The unbound MOZ DPF crystal structure was refined to a final Rfactor of 23.9% and Rfree of 25.5%, respectively. Ninety-five percent of residues were in preferred regions and 5% of residues in the allowed regions. In this structure, residues 270–273 were poorly defined in the electron density and therefore not modelled. MOZ DPF in complex with H3K14ac was refined to a final Rfactor of 21.9% and Rfree of 26.6%. In the final model, 95% of residues were in the preferred regions and 5% of residues were in the allowed regions of the Ramachandran plot. All steps of refinement were carried out using the PHENIX suite using a maximum likelihood target and non-crystallographic symmetry (NCS) restraints where applicable (23). After each round of refinement electron density maps were inspected visually and the models manually adjusted in COOT (22). The geometry and overall quality of the structures was assessed using MOLPROBITY (24).

## RESULTS

### The MOZ DPF binds H3 and H4 N-terminal tails and samples their PTM status

MOZ is a multidomain chromatin-associated protein containing a lysine acetyltransferase (MYST) domain, and an adjacent tandem DPF domain, as shown schematically in Figure 1A. Using a series of GST-MOZ constructs (Figure 1A; Supplementary Figure S1A), *in vitro* binding assays established that the DPF domain of MOZ interacts directly with histone H3 (Supplementary Figure S1B). Peptide surrogates demonstrated that this interaction is mediated by the H3 N-terminal tail, as MOZ DPF showed a robust interaction with H3 (1–21) (Figure 1B), but not H3 (22–44) (Supplementary Figure S1C). Association of MOZ DPF with H3 tail peptides was reduced by mono- or dimethylation of H3K4 and completely blocked by H3K4me3, whereas binding to peptides containing methylated forms H3K9 was tolerated (Figure 1B; Supplementary Figure S1D). We did not detect binding to peptides containing H3K27me1 or H3K36me1, consistent with the lack of interaction of MOZ DPF with this region of H3 (Supplementary Figure S1C and D). Western blots confirmed that the MOZ DPF can associate with H3 histones containing the repressive modification H3K9me3, but not H3K4me3 (Supplementary Figure S1E). Consistent with this result, recombinant full-length FLAG-MOZ protein, or the N-terminal portion of MOZ that is retained in leukaemogenic fusion proteins (FLAG-MOZ-N: 1-1117) (10) showed robust binding to unmodified H3 or H3K9me3 peptides, but not H3K4me3 (Supplementary Figure S1F). These binding patterns are consistent with other recent studies (17,18) and indicate a role for the DPF in sampling H3K4 methylation status, whereas H3K9 methylation did not appear to have a major influence on the MOZ DPF/H3 interaction interface.

The MOZ DPF domain also displayed a weaker ability to bind the H4 (2–24) N-terminal tail peptide, but not when the tail peptide was hyperacetylated (Figure 1B; Supplementary Figure S1G). Acetylation of H4K5, H4K8, H4K12 or H4K16, alone or in combination, completely abrogated binding to MOZ proteins (Supplementary Figure S1G). These results demonstrate an important difference between MOZ and DPF3b/BAF45c protein, as the latter was reported to show enhanced binding to H4 when K16 is acetylated (15).

In contrast to H4 acetylation, H3 tail peptides containing H3K9/14Ac showed enhanced recruitment of the MOZ DPF (Figure 1B), and further analysis indicated that acetylation of H3K14 stabilizes the binding of the H3 tail to MOZ DPF (Figure 1C). To further support these *in vitro* binding data, FLAG-MOZ protein was transiently expressed in U2OS cells for immunofluorescent staining. FLAG-MOZ showed a typical punctate nuclear stain as previously reported (10,13) and showed substantial co-localization with pan-acetyl histone H3 and H3K9me3 staining patterns (Supplementary Figure S2A). However, FLAG-MOZ foci overlapped poorly with H3K4me3 staining. Cross-linked chromatin was prepared from K562 leukaemic cells, which express high levels of MOZ, and subjected to immunoprecipitation with α-MOZ antibody or IgG control. As shown in Supplementary Figure S2B, western blots confirmed that MOZ-bound chromatin is enriched in H3K14Ac, but not H3K4me3. Taken together, these results indicate that MOZ associates with chromatin containing distinctive histone PTM signatures, i.e being enriched where there is acetylated H3, but largely excluded from H3K4me3-enriched chromatin. Thus, while acetylation of H3K14 stabilizes interaction of MOZ with H3, H4 acetylation may have opposing effects on MOZ recruitment. Consistent with these results, the DPF domain of MORF/MYST4 showed highly similar H3 PTM binding preferences (Supplementary Figure S1H).

To assess the contribution of the individual PHD domains of MOZ in binding to histone tails, we generated GST-PHD1 and GST-PHD2 constructs (Supplementary Figure S1A). The PHD1 and PHD2 subdomains displayed differential histone binding properties. PHD1 showed little interaction with any H3 peptides, but was capable of binding to unmodified H4 N-terminus (Supplementary Figure S1I). As observed for both the MOZ DPF domain and full-length MOZ, acetylation of H4 was incompatible with binding to PHD1 (Supplementary Figure S1I). PHD2 alone showed strong binding to unmodified H3 and H4, but not acetylated H4 peptides, but in contrast to the composite DPF, PHD2 also showed substantial binding to peptides containing H3K4me3 (Supplementary Figures S1I and J). Moreover, no enhanced binding to H3K14Ac peptides was observed (Supplementary Figure S1I). This indicates that while PHD2 domain can interact with the H3 tail, both PHD fingers are required to set the non-permissive selectivity of MOZ for H3K4me3, and to stabilize binding to H3K14Ac. Thus, the composite DPF is required for the ability of MOZ to sample H3K4 methylation and H3K14 acetylation status.

### A novel function for MOZ DPF in promoting acetylation of H3K14 and H3K9

The ability of the MOZ DPF to bind to H3 and H4 N-terminal tails dependent on the modification status suggested that the DPF functions in substrate selection, and thus might cooperate with the MYST acetyltransferase domain to promote histone acetylation. Time course acetylation assays revealed that the initial rate of acetylation of core histones by DPF-MYST was at least 8-fold faster than the MYST domain alone (Figure 1D). Peptide acetylation assays confirmed that the DPF-MYST construct can acetylate the H3 N-terminus (1–21), and immunoblots using H3 acetylation-specific antibodies confirmed substantial increases in acetylated H3K9 and H3K14 in the presence of GST-MOZ DPF-MYST, but not GST control (Figure 1E). Moreover, pre-acetylation of H3 at H3K9 and H3K14 resulted in substantially reduced or negligible levels of acetylation, respectively, as compared with unmodified H3 peptide, confirming that H3K14 and H3K9 are MOZ acetylation targets (Figure 1F).

The DPF-MYST construct was also capable of acetylating the H4 N-terminal tails but not H3 (21–44) (Figure 1G). Remarkably, H3 peptides containing H3K4me3 were not capable of being acetylated by DPF-MYST (Figure 1G), despite the availability of unmodified H3K9 and H3K14. In contrast, H3K9me3 showed only a partially reduced level of acetylation compared with unmodified H3 (1–21), owing to the availability of H3K14 for acetylation. These results highlight the key role of H3K4 in regulating the formation of MOZ/H3 complexes, and identify H3K14 and H3K9 as acetylation targets of MOZ. To investigate the effect of H3 acetylation on recruitment of the DPF-MYST, binding assays were performed in the presence of acetyl CoA. As shown in Figure 1H, acetyl CoA enhanced the binding of GST-DPF-MYST to immobilized H3 tail peptide, indicating that acetylation of the H3 N-terminal tail stabilizes the interaction of MOZ and H3. These results support the hypothesis that the MOZ DPF functions in binding to H3, thus facilitating its acetylation. In contrast, trimethylation of H3K4 prevents interaction of MOZ with H3 and hampers H3K14/K9 acetylation.

### The MOZ DPF induces a unique α-helical conformation in the H3 tail

To understand the molecular basis of H3 recognition by MOZ and the consequences of H3K9 and H3K14 acetylation, we determined crystal structures spanning MOZ DPF (194–323) alone and in complex with a series of histone H3 peptides (1–21), unmodified or acetylated at H3K9 or H3K14 to 3.0 Å, 1.6 Å, 1.6 Å and 2.5 Å resolutions, respectively. The DPF–H3 complex structure was solved by SAD using the anomalous signal from the zinc atoms and served as a search model in the structure determination of the DPF in isolation and in complex with H3K9ac and H3K14ac by molecular replacement. Data collection and refinement statistics are summarized in Table 1.

**Figure 2.**
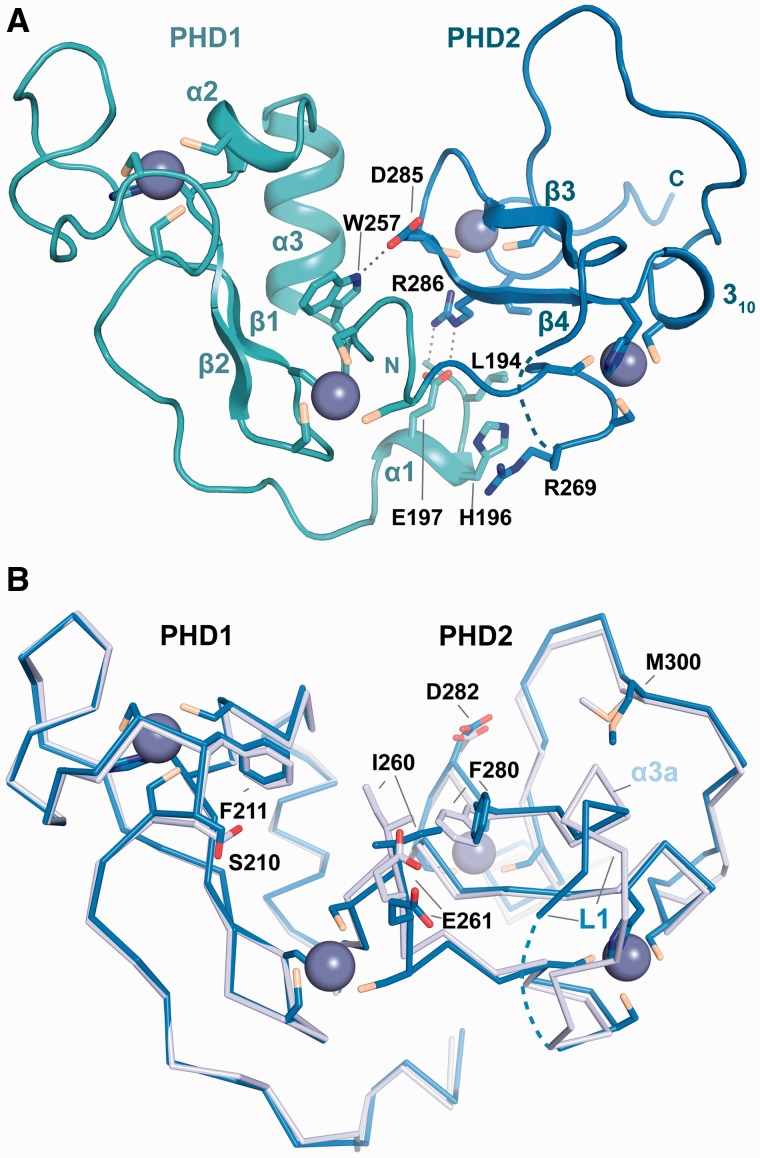
Structure of the MOZ DPF domain. (**A**) Crystal structure of MOZ DPF spanning residues L194-G316 with the zinc atoms shown as grey spheres and the secondary structure elements indicated. The PHD1 and PHD2 subdomains are coloured in turquoise and blue, respectively. Residues engaged in interactions with the N-terminal helix α1 and PHD2 as well as a direct hydrogen bonding interaction between PHD1 and PHD2 are labelled and shown in stick representation. (**B**) Superposition of ribbon representations of the unbound MOZ DPF domain (blue) and the MOZ DPF domain as observed in complex with unmodified H3 (light blue); H3 is not shown for clarity. Key residues that undergo conformational changes are shown in stick representation and are labelled. Note that PHD2 is more affected by the interaction with H3 with larger changes on binding observed. Linker L1 that precedes β3 is partly disordered and the H3K4 binding pocket is partially occluded in the unbound structure.

The structure of MOZ DPF shows the typical globular domain of a DPF with PHD1 and PHD2 each harbouring two zinc atoms coordinated by C4-H-C3 motifs in cross-brace topology (Figure 2A). In our structures, residues L194–D199 at the N-terminal boundary of PHD1 form a helix (denoted as α1) that folds back onto the DPF core and interacts with the PHD2 subdomain through salt bridge interactions between E197 and R286, hydrophobic contacts engaging L194 and I309 and interactions between the H196 and R269 side chains (Figure 2A). Other interactions between the PHD1 and PHD2 subdomains are observed, including a hydrogen bond between W257 and D285 (Figure 2A). On H3 binding, subtle conformational changes in the DPF domain occur in regions of direct contact with H3, including a side chain rotation of F280 and changes in a linker region (designated L1), which harbours a helical turn in the H3 complex (A275-N277; labelled as α3a in Figure 2B) but not in the unbound DPF structure.

**Figure 3.**
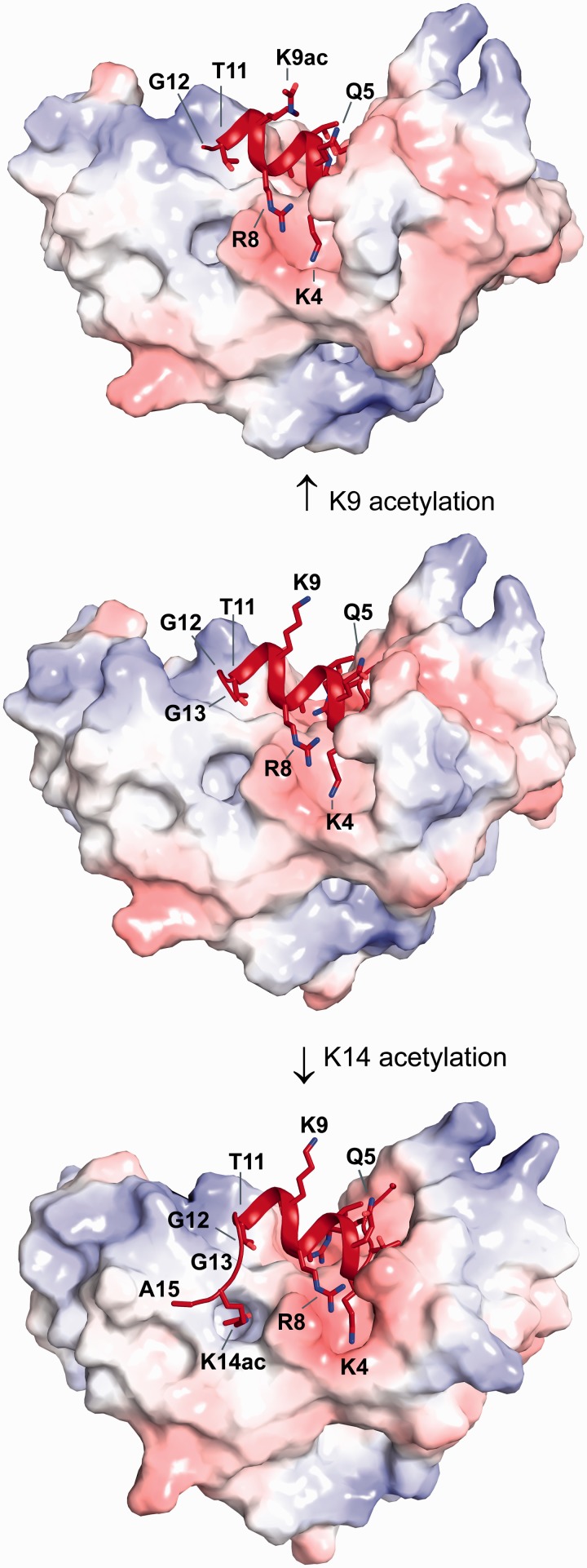
Structural basis of H3, H3K9ac and H3K14ac recognition by MOZ. Surface representation of the MOZ DPF domain coloured according to electrostatic potential in complex with unmodified H3 (middle), H3K9ac (top) and H3K14ac (bottom) in red cartoon representation. Please note the exposed position of the H3K9 side chain, the preservation of the helical conformation that is independent of acetylation of either K9 or K14 and the GG hinge that mediates conformational changes to allow K14ac to bind to a pocket on the DPF domain.

**Figure 4.**
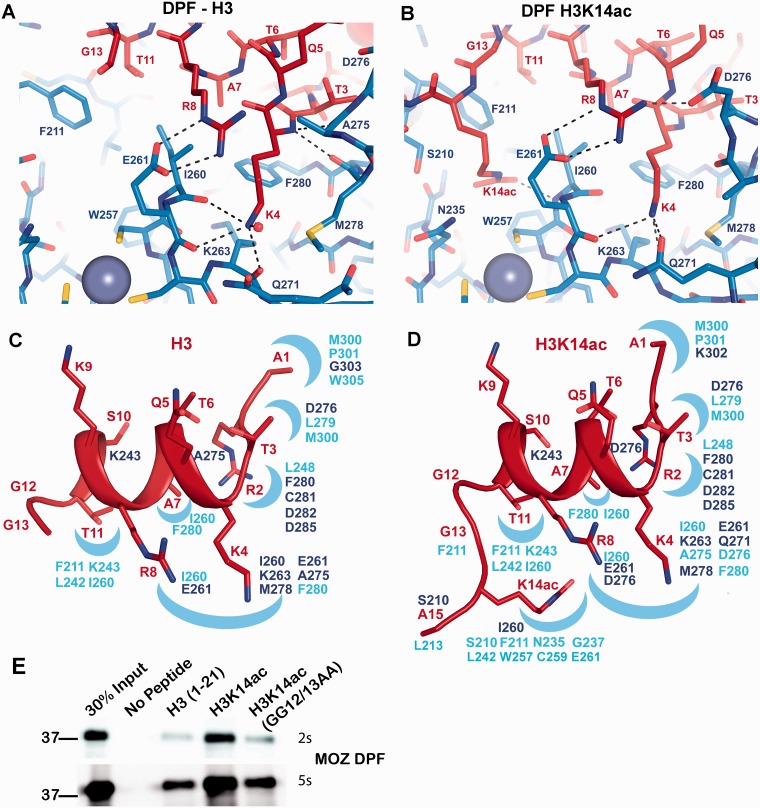
Interactions of H3 and H3K14ac with the MOZ DPF. (**A, B**) Close up view of complex crystal structures in stick representation, highlighting the interactions with key residues labelled; blue denotes for MOZ DPF residues and red for H3 or H3K14ac. The DPF–H3 complex is shown in (A) and the DPF–H3K14ac complex in (B). Plausible hydrogen bonding interactions are indicated by dashed lines. Zinc atoms are shown as grey spheres. (**C**) H3 N-terminal tail structure as seen in the complex with MOZ DPF and (**D**) H3K14ac structure as seen in the complex with MOZ DPF depicted in red. Binding pockets on the DPF surface are schematically indicated as blue crescents. Corresponding interacting residues involved in hydrogen bonding interactions (dark blue) or hydrophobic contacts (light blue) are indicated. (**E**) Binding of GST-MOZ DPF to histone H3 tail peptides as indicated, i.e. unmodified H3, H3K14ac or H3K14ac peptide in which the GG hinge is mutated. Two exposures are shown (2 or 5 s) to highlight the differential binding.

**Figure 5.**
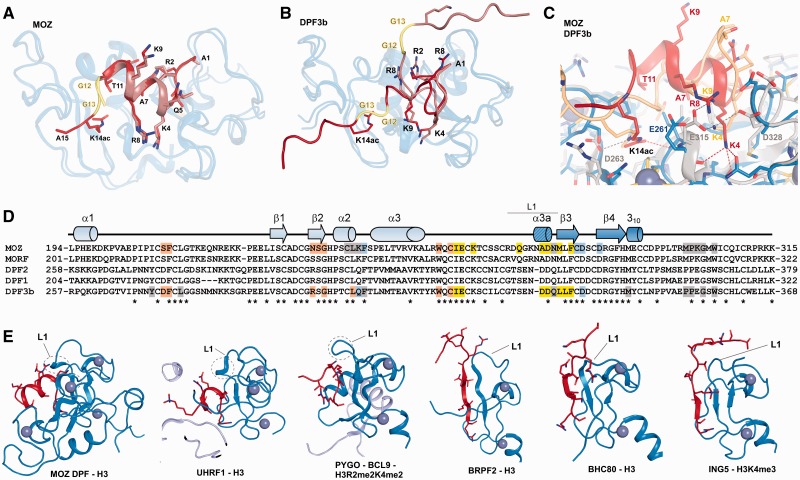
Comparison of MOZ DPF–H3 with other PHD–H3 complexes. (**A**) Superposition of MOZ DPF (blue; semi-transparent) in complex with H3 (brown) and H3K14ac (red). The GG hinge region that mediates the insertion of K14ac into the respective binding pocket is highlighted in yellow. (**B**) Superposition of DPF3b in complex with unmodified H3 [PDB code: 2KWK (15)] and with H3K14ac [PDB code: 2KWJ (15)] depicted in same colour coding. In the DPF3b complex structures, the H3 conformation is extended. (**C**) Close up view of a superposition between MOZ DPF–H3K14ac (blue; red) and DPF3b–H3K14ac (grey; beige) (**D**) Sequence alignment with MOZ DPF with other DPF domain proteins. Secondary structure elements observed in our structures are shown above the alignment and MOZ residues that are engaged in H3 recognition are highlighted. Light blue denotes residues involved in H3R2 interactions, yellow for H3K4 and orange for H3K14ac interactions. Residues engaged in other interactions are depicted in grey. Colour coding is the same for DPF3b. (**E**) Comparison of H3 conformations in complex with assorted PHD domains. Cartoon representations of a number of DPF and PHD domains depicted in blue and H3 tail peptides depicted in red. From left to right: MOZ DPF-H3; UHRF1-H3 {PDB code 3ASK (35); PYGO-BCL9-H3R2me2K4me2 [PDB code: 2VPG; (33)]; BRPF2-H3 [PDB code: 2L43; (45)]; BHC80-H3 [PDB code 2PUY; (46)]; ING5-H3K4me3 [PDB code 3C6W; (47)}. Circled is a turn feature in the linker region L1 that may contribute to induction of helical conformation in H3 when interacting with PHD domains such as seen in MOZ, UHRF1 and potentially PYGO-BCL9.

Unexpectedly, the H3 tail adopts extensive α-helical conformation in complex with MOZ DPF (Figures 3 and 4; Supplementary Figure S4A) in contrast to the extended conformation of H3 peptides observed in other complexes (see Figure 5E). This helix encompasses residues H3K4-T11 and spans across the DPF domain (Figure 3). As a consequence, these residues occupy different spatial positions with side chains of H3K4, H3A7, H3R8 and H3T11 protruding from one side of the helix, while the H3Q5, H3T6, H3K9 and H3S10 side chains occupy the opposite side (Figures 3, 4C and D; Supplementary Figure S3A). Overall, the MOZ DPF-H3 interface area is characterized by distinct binding pockets that exhibit negative electrostatic potential (Figure 3) to accommodate the positively charged H3 lysine and arginine residues. Hydrogen bonds and salt bridges dominate the interface although hydrophobic interactions also contribute (Figure 4A and C; Supplementary Figure S4C). The side chain of H3A1 inserts into a hydrophobic pocket formed by residues M300, P301 and W305 while the N-terminal amine forms a hydrogen bond with the main chain carbonyl of G303 (Supplementary Figure S4C). H3R2 engages in a network of hydrogen bonding and electrostatic interactions with two aspartate residues, D282 and D285, the main chain carbonyl of C281 and the main chain amine of F280 (Supplementary Figure S4C). H3T3 forms hydrophobic contacts with L279 and M300 and the hydroxyl group forms a hydrogen bond with D276 in the α3a helix induced by complex formation (Figure 4C; Supplementary Figure S4C).

The side chain of H3K4 inserts into a pocket on the surface of MOZ DPF (Figures 3 and 4C) that is partially occluded by residues from the L1 linker in the unbound DPF crystal structure (Figure 2B). Interestingly, H3K4 engages in direct hydrogen bonding interactions with the main chain carbonyls of I260, E261 and K263 (Figure 4A), an atypical binding mode for H3K4 in comparison with other PHD complexes that was also not observed using the shorter construct (18). A water molecule also coordinates the Nε-amino group and hydrophobic contacts occur between A275 and H3K4. H3R8 also protrudes towards the H3K4 pocket (Figure 4A and C), with the guanidinium group forming salt bridge interactions with E261. In addition, the main chain amine of H3Q5 forms a hydrogen bond with the carbonyl group of A275, whereas H3A7 forms hydrophobic contacts with I260 and F280. The main chain carbonyl of H3S10 engages in a hydrogen bond with K243. Finally, H3T11 occupies a hydrophobic pocket formed by F211, L242, I260 and K243. H3G12 and H3G13 break the alpha helical conformation (Figure 4C). No interpretable density is visible beyond H3G13, indicating that H3 residues 14–21 are flexible and do not interact with the DPF domain. The binding mode of H3K4 (Figure 4A) provides a rationale for the selectivity of MOZ against H3K4me3, as in addition to the steric implications, the trimethyl ammonium moiety would be unable to form hydrogen bonds with the K4 binding pocket. Although mono- or dimethylated forms of H3K4 would still be capable of acting as hydrogen bond donors to carbonyl groups, the reduced binding observed is likely due to steric effects.

### A double-glycine hinge mechanism facilitates H3K14ac docking with the DPF

In this novel H3 binding mode, the side chains of H3Q5, H3T6, H3K9 and H3S10 may be amenable to modification by other histone modifying enzymes. In particular, the side chain of H3K9 is orientated away from the DPF in a position ideal to allow its modification, e.g. acetylation by the MYST domain, consistent with our data (Figure 1D and E) and other studies (25,26). Based on the biochemical assays (Figure 1C) and our MOZ DPF–H3 complex structure (Figure 3) we reasoned that H3K9 modification would have little impact on the helical structure of the N-terminal tail in complex with MOZ. To test this hypothesis, we crystallized the MOZ DPF with a peptide harbouring an acetylation modification on H3K9. The structure confirmed our hypothesis and shows that the side chain of H3K9ac is highly flexible consistent with its location pointing away from the DPF domain, while the helical conformation of the tail is preserved (Figure 3; Supplementary Figure S3B).

To investigate the impact of H3K14ac on the helical structure of the H3 tail, we crystallized a MOZ DPF–H3K14ac complex. The structure revealed that the helical conformation of H3K4-T11 is preserved (Figure 3; Supplementary Figure S4B) and the majority of interactions in this region of H3 are unaltered. However, the interface area (844.6 Å^2^) is increased in comparison with the DPF-H3 structure (695 Å^2^), revealing the molecular basis for H3K14ac recognition by MOZ. Importantly, a conformational change mediated through H3G12-G13 allows the acetylated H3K14 side chain, now devoid of charge, to insert into a preformed pocket on the DPF surface (Figure 3; Supplementary Figures S3C and S4B). This pocket (lined by S210, F211, N235, L242, W257, C259 and I260) is mainly hydrophobic and exhibits a weak positive electrostatic potential, rendering it unfavourable for unmodified K14 interaction (Figures 3 and 4D). The acetyl group of H3K14ac forms a hydrogen bond with the main chain amine of I260, revealing that I260 makes contacts with both H3K14ac and H3K4. While H3A15 engages in a hydrophobic contact with L213, residues C-terminal to this are not visible in the electron density, indicating that they are not involved in interactions with the DPF domain. The observed structural changes between H3 and H3K14ac in complex with the DPF reveal a mechanism for modulating H3 interactions with MOZ. To validate the requirement of the double glycine (GG) hinge for docking of H3K14Ac, we used a H3 peptide in which G12 and G13 were substituted with alanine residues. As shown in Figure 4E, while MOZ-DPF showed enhanced binding to the H3K14ac peptide as compared with unmodified H3 tail, this enhanced interaction was not observed using the GG12/13AA peptide, which bound the DPF to a similar level as unmodified H3. This result highlights the importance of the GG hinge to enable engagement of the H3K14Ac by MOZ. The results also suggest for the first time a functional role for H3 tail residues G12 and G13 in epigenetic signalling.

### H3 tail recognition by MOZ and DPF3b/BAF45c

Aside from MORF, the MOZ DPF domain shares sequence homology with the BAF45 proteins (also known as DPF1–3) (Figure 5D), which are key components of the mammalian Switch/Sucrose Non fermentable (SWI/SNF) or BAF complexes (27). The DPF domain of DPF3b/BAF45c [Root Mean Square Deviation (RMSD) = ∼1.9 Å; Z score = 13.7; sequence identity of 52% (28)] is the only other DPF domain for which both unmodified H3 and H3K14ac complex structures are currently available for comparison (15). While the MOZ structure contains an additional N-terminal helix (α1), DPF3b contains an extra helix at its C-terminus (Figure 5B). The N-terminal anchor of H3 (residues A1-R2-T3) adopts similar conformations in both MOZ and DPF3b structures. However, a major difference arises in the way H3K4 is recognized. H3K4 forms hydrogen bonds/salt bridges with two acidic residues in DPF3b, E315 (E261 in MOZ DPF) and D328 (whose counterpart is A275 in MOZ) (Figure 5C) as opposed to interactions with main chain carbonyls in MOZ. In addition, the L1 region in DPF3b is shorter by one residue. Strikingly, none of the reported DPF3b complex structures reveal any helical conformation of H3 peptides (Figure 5B) (15). As a consequence, H3K9 protrudes into the H3K4 pocket by interacting with E315 in DPF3b, whereas H3R8 occupies this position in the MOZ complexes (Figure 5C).

While H3 residues 4–13 adopt entirely different conformations and engage in different contacts with MOZ and DPF3b, H3K14ac occupies equivalent binding pockets, albeit involving different interactions (Figure 5C). In DPF3b, H3K14ac Nε forms a hydrogen bond with the side chain of D263 (PDB code 2KWJ) (15). In the MOZ–DPF complex, H3K14ac is within hydrogen bonding distance of MOZ I260, and engages in van der Waals contacts with S210 at the rim of the pocket. MOZ N235 and R289 in DPF3b occupy equivalent positions and both engage in hydrophobic contacts with H3K14ac. Thus, while the acetylated H3K14 occupies equivalent pockets within the PHD1 moieties of MOZ and DPF3b, different interactions are used to achieve this (Figure 5C). The alternative conformations of the H3 tail may reflect functional differences between DPF3b and MOZ. Unlike MOZ and MORF, BAF45/DPF proteins are not known to harbour any intrinsic enzymatic activity, and thus may function uniquely as histone PTM ‘reader’ components that can tether BAF complexes to appropriate chromatin targets. MOZ on the other hand, imposes a structural conformation on the H3 tail to facilitate its acetylation. This ability of MOZ to manipulate H3 tail conformation reflects its dual role in reading and modifying histone codes to drive developmental gene expression in haematopoietic and other tissues.

## DISCUSSION

While chromatin-associated proteins are clearly essential components of normal gene regulatory systems, relatively little is known of their impact on chromatin/histone structure.

Early observations from the Grunstein and Wolffe groups predicted that histone tails would adopt helical conformations when participating in macromolecular interactions (29,30). Helical propensity of the H3 tail is also detected by secondary structure prediction algorithms. However, until now, structural studies investigating H3 tail interactions with chromatin readers have generally observed extended conformations of the histone tail. A commonly observed binding mode involves the formation of antiparallel beta sheets between single PHD domains and H3 N-terminal residues (31,32), as seen in bromodomain PHD finger 2(BRPF2), BRAF35 HDAC Complex protein (BHC80) and inhibitor of growth family member 5 (ING5) complexes (Figure 5E). The predominantly helical H3 conformations seen in complex with MOZ DPF reported in this study are at present unique. This more compact conformation, in conjunction with a GG hinge, allows MOZ DPF to engage at least the first 15 residues of the K14 acetylated H3 tail. This H3 binding mode not only enables MOZ to sample multiple H3 PTMs, but also facilitates acetylation of H3, providing that the H3K4me3 mark is absent.

In comparison, single PHD domains typically recognize up to nine residues of an extended H3 tail (16). However, on close inspection of available structures, we noticed indications of potential helicity in a subset of H3 complex structures (Figure 5E). H3 residues 4–5 in complex with the PHD domain of Pygopus-BCL9 [PDB code: 2VPG, (33); Figure 5E] closely mimic the start of a helical turn and this is also seen in a double chromodomain H3 complex (PDB code: 2B2T) (34). Recently a single helical turn has been observed in complex with the PHD domain of the E3 ubiquitin ligase UHRF1 [PDB code 3ASK (35); Figure 5E]. Thus we noted that helical propensity of the H3 tail in these complexes appeared to correlate with differences in the L1 linker sequences of the reader module. A helical turn or loop in longer L1 linkers (such as α3a in MOZ; circled in Figure 5E) may induce a degree of helicity by ‘bending’ H3 tails. Alternatively, shorter L1 regions probably favour antiparallel sheet formation such as seen for BRPF2 (Figure 5E). These observations lead us to speculate that the L1 linker region may influence H3 conformation as highlighted in Figure 5E. Thus, partially helical conformations of the H3 tail may also be a feature of its recognition by other histone-binding domains awaiting structure elucidation.

Our structures provide novel insight into histone tail PTM sampling by a chromatin reader. Binding of MOZ DPF to the H3 tail is diminished by H3K4me3 (Figure 1B), but enhanced by H3 acetylation (Figure 1B and C), and the structures show the molecular detail of how H3K14 acetylation promotes further contacts within the complex through conformational changes in H3 (Figures 3 and 5A). A recently reported structure of a shorter MOZ DPF construct (residues 202–313) bound to H3 residues 1–7 (18) agrees with our findings with regard to how residues H3 A1-T3 engage with MOZ. However, the absence of the sequence comprising α1 helical region in the truncated MOZ DPF, and occupation of the H3K14Ac binding pocket by acetate from the crystallization conditions are likely to account for the otherwise major differences in H3 contacts observed, including the lack of helical structure in the H3 tail (Supplementary Figure S5).

**Figure 6.**
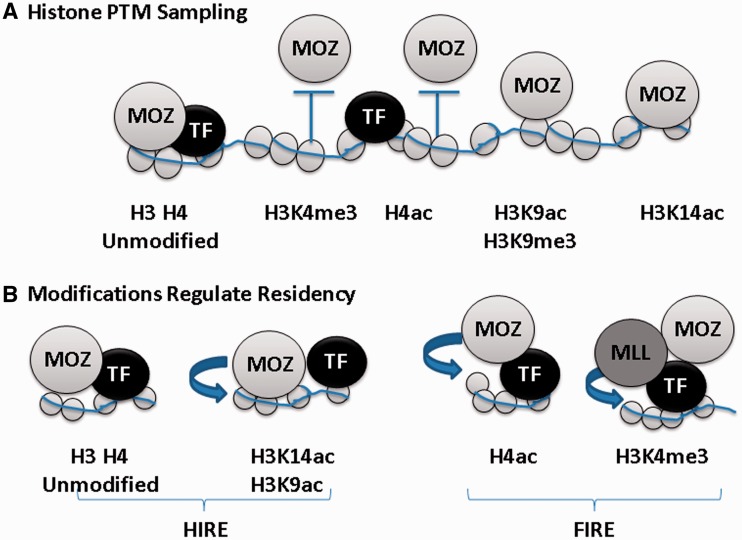
(**A**) Histone PTM sampling by MOZ. Schematic representation showing the recruitment of MOZ proteins to chromatin through complex formation with transcription factors (TFs) or by direct interaction of MOZ with histones. Unmodified H3 and H4 tails, or modifications such as H3K9ac or H3K9me3 are permissive for MOZ recruitment. MOZ recruitment is reinforced by H3K14 acetylation. However, H3K4me3 or H4 tail acetylation constitutes non-permissive PTMs that may repulse MOZ from chromatin. (**B**) Histone acetylation by MOZ regulates its occupancy on chromatin. PTM sampling by the MOZ DPF facilitates histone substrate selection for acetylation. Acetylation of H3K14 stabilizes residency of MOZ on chromatin, whereas acetylation of H4 by MOZ or other regulators is non-permissive for MOZ recruitment. Histone PTMs generated by other factors such as the MLL histone methyltransferase complex that catalyses H3K4 trimethylation, also impact on MOZ residency. Thus, chromatin modifying proteins generate ‘Hire’ and ‘Fire’ PTM signatures that regulate their interactions with chromatin.

While our structures reveal how acetylation of the H3 tail at K14 can stabilize the interaction with MOZ, acetylated H4 tail peptides failed to bind MOZ in *in vitro* binding assays (Figure 1B; Supplementary Figure S1G). These results suggest opposing effects of H3 and H4 acetylation on their interactions with MOZ as summarized in Figure 6. The orientation of H3K9 protruding away from the DPF domain suggests it is ‘presented’ for modification by the intrinsic MYST catalytic domain or other enzymes such as H3K9 methyltransferases present in MOZ complexes (36). We have also shown that acetylation of H3K9 does not impact on the H3 conformation observed in complex with the DPF domain. Similarly, the unique conformation of the H3 tail in complex with MOZ suggests that H3T6, H3R8, H3S10 or H3T11 may also be amenable to PTM sampling or poised for modification (Figure 4C and D).

In the unmodified H3-DPF complex, the region C-terminal to H3G13, including H3K14, is not in contact with the DPF and is therefore also potentially available for acetylation by the MOZ MYST domain. The acetylated H3K14 can dock with the MOZ DPF, as a consequence of the structural flexibility of the preceding GG hinge (Figure 5A) as confirmed by the binding assay showing that the GG hinge is required for enhanced binding of MOZ to H3K14Ac (Figure 4E). Interestingly, other GG motifs are present in the N-terminal tails of the core histones, where they may perform similar functions as structural ‘hinges’. These GG motifs also flank lysine residues that are important regulatory targets in chromatin such as H3K36, H4K8, H4K16 and H2AK9. Interestingly, mutations in the G34 residue in H3.3 variant histones have been discovered as key drivers in glioblastomas, although how this impacts on chromatin modification/remodelling remains to be determined (37,38). Thus, as in the case of MOZ and H3K14 acetylation, GG hinges may facilitate alternate conformations that modulate histone tail interactions with other chromatin regulators.

Our data reveal a role for MOZ DPF in selection of histone substrates for acetylation. While the MOZ MYST domain acetylates both H3 and H4 substrates *in vitro*, the rate of H3 acetylation is strongly enhanced by the presence of the DPF domain (Figure 1D). MOZ acetylates both H3K14 and H3K9 *in vitro* (Figure 1E and F) consistent with a report that ING5/MOZ/MORF complexes acetylate both H3 and H4 acetylation, and in particular target H3K14 (25). Interestingly, a recent study reported significant coincidence of H3K9ac and H3K14ac marks across the genome of ES cells, and showed that both modifications are associated with the promoters and enhancers of actively transcribed genes (39). The study also reported that H3K14ac marks a subset of genes that are poised for activation. ChIP-Seq studies will be required to identify MOZ binding sites across the genome and to assess whether these are enriched in H3K14ac or other histone PTMs. Such studies will help establish how composite PTMs can transduce their downstream functions.

The inability of MOZ to bind H3K4me3 correlates with its failure to acetylate H3K4me3 peptides (Figure 1G). Methylation of H3K4 plays a central role in the regulation of gene expression, as H3K4me3 is enriched within transcriptionally active regions of the genome. Trimethylation of H3K4 is catalysed by Mixed lineage leukaemia (MLL) complexes, which also contain the MYST family protein males absent on the first (MOF), responsible for H4K16 acetylation (40,41), and the combination of these PTMs promotes recruitment of the promotes recruitment of the nucleosome remodelling factor (NURF) chromatin remodelling complex. Interestingly, the BPTF subunit of the NURF complex binds efficiently to H3K4me3 and H4K16ac via its PHD and bromodomains, respectively (5), thus showing opposing histone binding preferences to the DPF domains of MOZ and MORF. However, MOZ and MORF can associate with complexes containing ING5 and BRPF1/2/3 proteins (42,43), which bind H3K4me3 and unmodified H3, respectively. Such differential binding specificities within multiprotein complexes may further facilitate co-regulator exchange on chromatin, as a consequence of sequential manipulation and recognition of histone PTMs.

Despite their sequence and structural similarity, MOZ and DPF3b exhibit major differences in how they recognize H3. Sequence variations in the H3K4 pocket are likely to be responsible for the different H3K4 binding modes (Figure 5C and D). Our results also indicate that acetylation has differential effects on binding of H4 to MOZ and DPF3b. DPF3b was reported to bind H4 tail peptides weakly compared with H3 peptides, although binding to H4 was reported to be substantially increased following K16 acetylation (15). In our hands, the relatively weak binding of MOZ DPF to H4 tail peptides was prevented by single or multiple acetylation at K5, K8, K12 or K16 (Figure S1G). Thus, high levels of acetylated H4 in our core histone preparations may also contribute to failure to detect H4 binding to MOZ *in vitro* (Supplementary Figure S1B).

The residence of MOZ, MORF and other epigenetic regulators in large macromolecular complexes provides a plausible model for how dynamic PTM signalling could be achieved through sequential binding and release of chromatin recognition modules and associated enzymatic domains within the complex. By sequential modification of H3 and H4 substrates, MOZ might dictate its own residency on chromatin, in combination with other chromatin regulators as summarized in Figure 6. Our findings that MOZ significantly alters H3 tail conformation to sample and modify the histone code provides new insight into these functions of chromatin regulators. The successful targeting of epigenetic regulators by small molecules in preclinical models of leukaemia (44,48,49), underlines the potential for translating chromatin biology into new therapeutic strategies. Thus, in addition to uncovering novel aspects of chromatin regulator function, our findings will aid the development of targeted strategies for MOZ-associated leukaemias.

## ACCESSION NUMBERS

Model coordinates and structure factor files were deposited under accession codes 4LJN (MOZ DPF), 4LK9 (MOZ DPF in complex with H3), 4LKA (MOZ DPF in complex with H3K9ac) and 4LLB (MOZ DPF in complex with H3K14ac) in the Protein Data Bank.

## SUPPLEMENTARY DATA

Supplementary Data are available at NAR Online.

Supplementary Data
