# Supplementary material for: The double PHD finger domain of MOZ/MYST3 induces α-helical structure of the histone H3 tail to facilitate acetylation and methylation sampling and modification
Source: Nucleic Acids Res. 2013 Oct 21;42(2):822–35. doi: 10.1093/nar/gkt931 (PMC3902925; doi:10.1093/nar/gkt931)
Supplement: Supplementary Data [file supp_42_2_822__index.html]

The double PHD finger domain of MOZ/MYST3 induces α-helical structure of the histone H3 tail to facilitate acetylation and methylation sampling and modification — The double PHD finger domain of MOZ/MYST3 induces α-helical structure of the histone H3 tail to facilitate acetylation and methylation sampling and modification — Supplementary Data 

# The double PHD finger domain of MOZ/MYST3 induces α-helical structure of the histone H3 tail to facilitate acetylation and methylation sampling and modification

## Supplementary Data

files

**Files in this Data Supplement:**

- Supplementary Data - pdf file
