# Supplementary material for: The double PHD finger domain of MOZ/MYST3 induces α-helical structure of the histone H3 tail to facilitate acetylation and methylation sampling and modification
Source: Nucleic Acids Res. 2013 Oct 21;42(2):822–35. doi: 10.1093/nar/gkt931 (PMC3902925; doi:10.1093/nar/gkt931)

Dreveny, Deeves et al., Supplementary Figures

FIGURE S1

Binding of MOZ to histones H3 and H4 is dependent on their acetylation and methylation status.

(**A**) SDS-PAGE and Coomassie staining was used to verify normalized amounts of each GST-fusion protein for comparison in binding and acetylation experiments. (**B**) Pulldown assays using immobilized GST or GST-DPF and calf thymus extracted core histones. Retention of specific histones was detected by western blotting. (**C, D, G, I, J**) Pulldown assays showing association of GST-MOZ proteins with immobilized biotin-conjugated H3 and H4 peptides containing modifications, as indicated. To detect the weaker H4 binding longer exposures relative to H3 binding assays were used. (**E)** Western blots showing association of H3K4me3 or H3K9me3-labelled histones with immobilized GST-MOZ DPF. (**F**) Association of *in vitro* translated 35[S]-labelled FLAG-MOZ or FLAG-MOZ-N (1-1117) proteins with immobilized biotin-conjugated H3, H3K4me3 or H3K9me3 peptides. (**H**) Pulldown assays showing association of GST-MORF DPF with immobilized H3 and H4 peptides containing modifications, as indicated. Note that for clarity, gel images in (**I**) have been cropped to allow them to be presented in a logical order for comparison.

FIGURE S2

Co-localisation of MOZ with acetylated H3, and exclusion of MOZ from H3K4me3 enriched chromatin.

(**A**) U2OS cells were seeded onto coverslips and transfected after 24 hours with expression plasmids encoding FLAG-MOZ. After 48 hours post-transfection, cells were fixed in 4%

paraformaldehyde, permeabilised and stained with mouse anti-FLAG antibodies (red) or rabbit antibodies specific against H3K4me3, H3K9me3 or pan-acetyl H3 as indicated (green). Secondary staining was achieved using anti-mouse Alexa594 and anti-rabbit Alexa488 secondary antibodies. Merged images indicate the degree of colocalisation of stained foci. Nuclear DNA was stained using Hoechst 33258 (blue).

(**B**) Chromatin IP assays showing that MOZ-associated chromatin is enriched in H3K14Ac but not H3K4me3. Crosslinked chromatin was prepared from K562 cells, immunoprecipitated with α-MOZ (KAT6A) antibody or IgG control. After reversal of crosslinks and SDS PAGE, western blots were used to detect H3K14Ac and H3K4me3.

FIGURE S3

Electron density of H3, H3K9ac and H3K14ac peptides

(**A**) Stereo-image of sigmaA weighted 2Fobs-Fcalc electron density map of H3 contoured at one sigma level (depicted in red) in complex with the MOZ DPF domain (depicted in blue). Hydrogen bonding interactions involving H3K4 and H3R8 are shown as black dashed lines.

(**B**) Stereo-image of sigmaA weighted 2Fobs-Fcalc electron density map of H3K9ac contoured at one sigma level (red) in complex with the MOZ DPF domain (blue). Hydrogen bonding interactions involving H3K4 and H3R8 are shown as black dashed lines.

(**C**) Stereo-image of sigmaA weighted 2Fobs-Fcalc electron density map of H3K14ac contoured at one sigma level (red) in complex with the MOZ DPF domain (blue). Hydrogen bonding interactions involving K4, R8 and K14ac are shown as black dashed lines.

FIGURE S4.

Alternative view of H3 interactions with MOZ DPF

(**A,B**) Surface representation of the MOZ DPF domain colored according to electrostatic potential in complex with (**A**) unmodified H3 or (**B**) H3K14ac in red cartoon representation. (**C,D**) Close-up view of complex structures in stick representation highlighting the interactions with key residues labeled; blue denotes for MOZ DPF residues and red for H3 or H3K14ac. The DPF-H3 complex is shown in (**C**) and the DPF-H3K14ac complex in (**D**). Plausible hydrogen bonding interactions are indicated by dashed lines. Zinc atoms are shown as gray spheres.

FIGURE S5

Superposition of MOZ DPF structures

Superposition of MOZ DPF crystal structure (blue) in complex with residues 1-15 of H3K14ac (red) with crystal structure of MOZ DPF - H3 (residues 1-7) obtained in the presence of H3K14ac peptide and acetate (PDB code: 3V43 (Ref. 18); DPF domain: light blue; H3 peptide and acetate observed in the structure: orange).

FIGURE S1

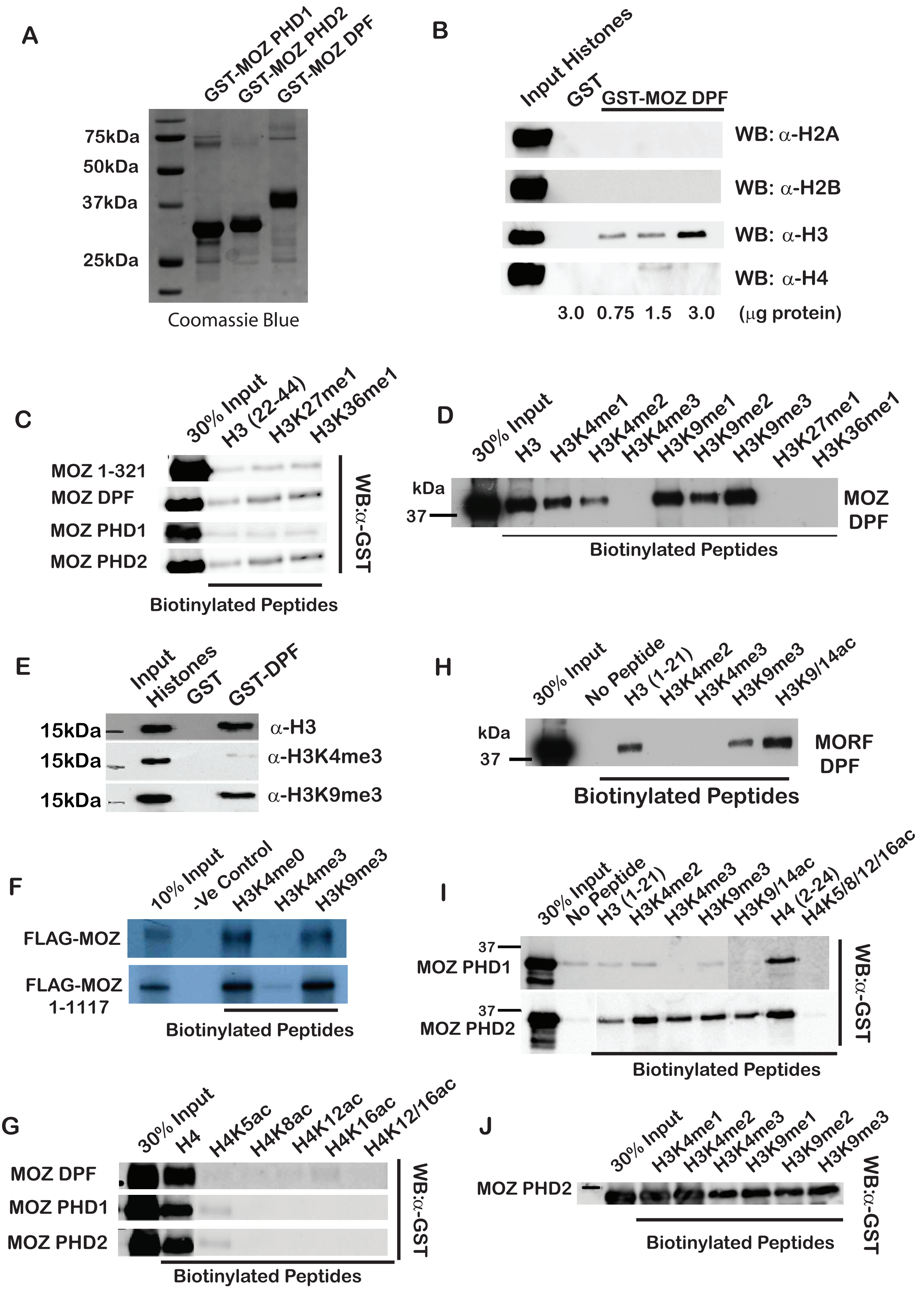

A
GST-MOZ PHD1
GST-MOZ PHD2
GST-MOZ DPF
75kDa
50kDa
37kDa
25kDa
Coomassie Blue
B
Input Histones
GST
GST-MOZ DPF
WB: α-H2A
WB: α-H2B
WB: α-H3
WB: α-H4
3.0 0.75 1.5 3.0 (μg protein)
C
30% Input
H3 (22-44)
H3K27me1
H3K36me1
MOZ 1-321
MOZ DPF
MOZ PHD1
MOZ PHD2
WB:α-GST
Biotinylated Peptides
D
30% Input
H3
H3K4me1
H3K4me2
H3K4me3
H3K9me1
H3K9me2
H3K9me3
H3K27me1
H3K36me1
kDa
37
MOZ DPF
Biotinylated Peptides
E
Input Histones
GST
GST-DPF
15kDa
15kDa
15kDa
α-H3
α-H3K4me3
α-H3K9me3
H
30% Input
No Peptide
H3 (1-21)
H3K4me2
H3K4me3
H3K9me3
H3K9/14ac
kDa
37
MORF DPF
Biotinylated Peptides
F
10% Input
-Ve Control
H3K4me0
H3K4me3
H3K9me3
FLAG-MOZ
FLAG-MOZ 1-1117
Biotinylated Peptides
I
30% Input
No Peptide
H3 (1-21)
H3K4me2
H3K4me3
H3K9me3
H3K9/14ac
H4 (2-24)
H4K5/8/12/16ac
37
MOZ PHD1
37
MOZ PHD2
WB:α-GST
Biotinylated Peptides
G
30% Input
H4
H4K5ac
H4K8ac
H4K12ac
H4K16ac
H4K12/16ac
MOZ DPF
MOZ PHD1
MOZ PHD2
WB:α-GST
Biotinylated Peptides
J
30% Input
H3K4me1
H3K4me2
H3K4me3
H3K9me1
H3K9me2
H3K9me3
MOZ PHD2
WB:α-GST
Biotinylated Peptides

FIGURE S2

A

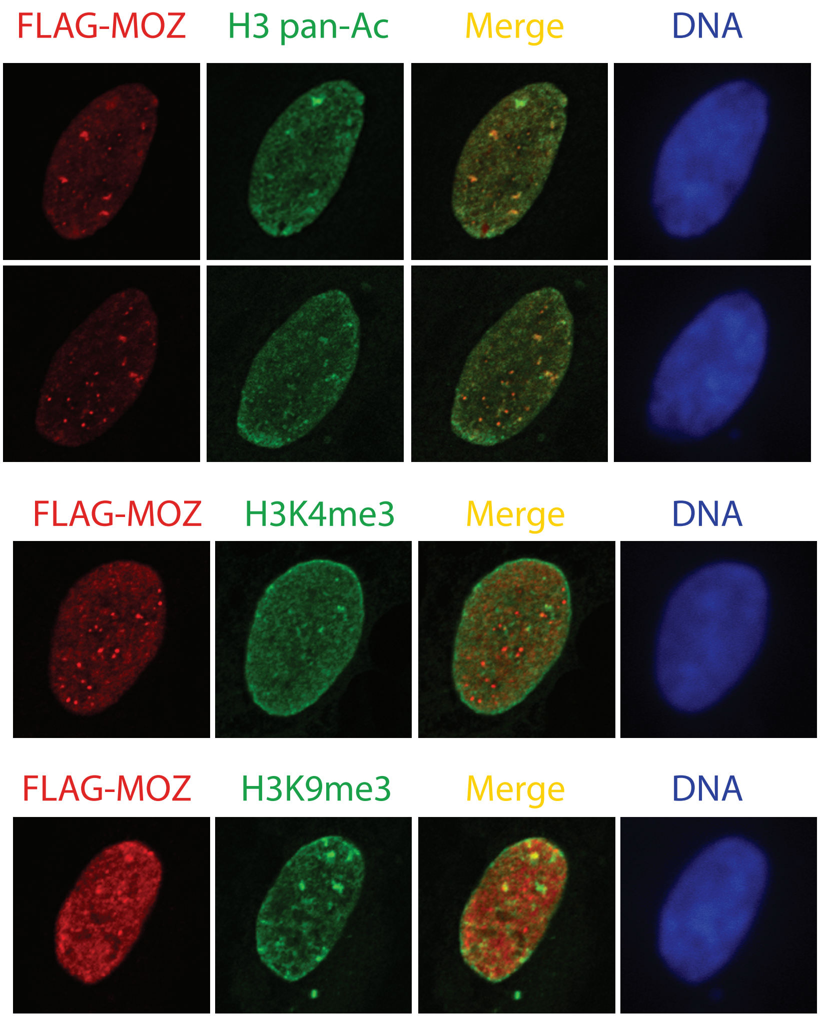
FLAG-MOZ
H3 pan-Ac
Merge
DNA
FLAG-MOZ
H3K4me3
Merge
DNA
FLAG-MOZ
H3K9me3
Merge
DNA

B

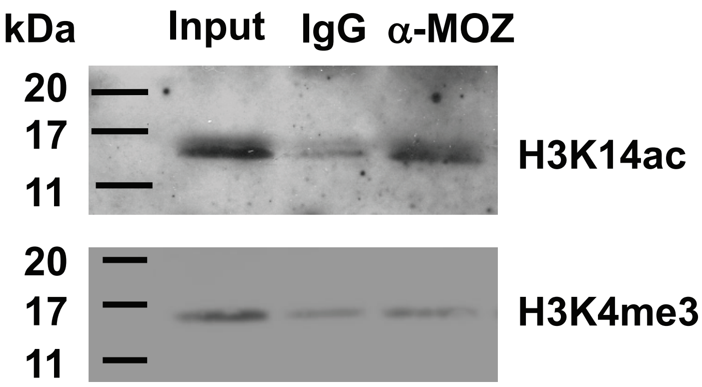
kDa
Input
IgG
α-MOZ
20
17
11
H3K14ac
20
17
11
H3K4me3

FIGURE S3

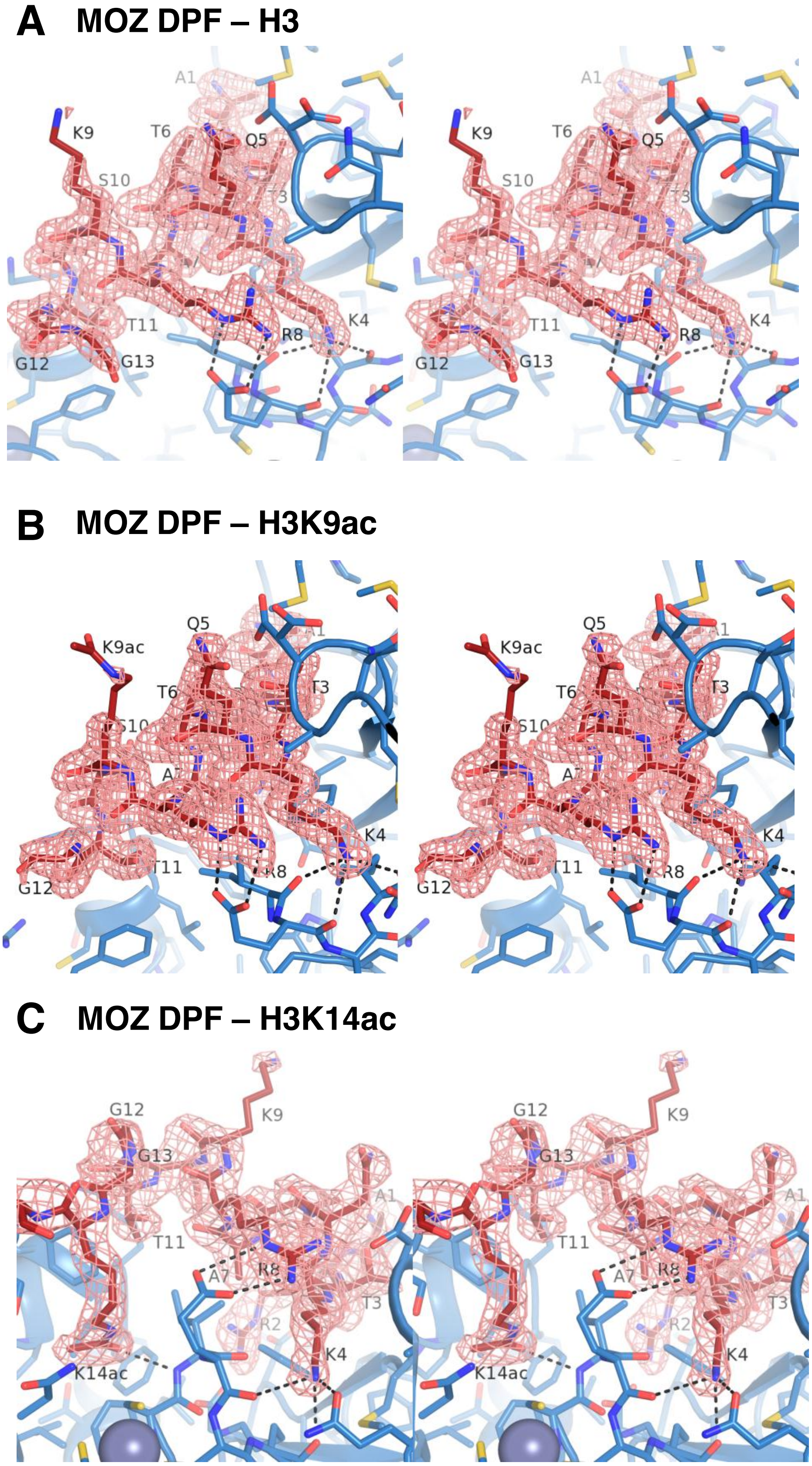
A
MOZ DPF – H3
A1
K9
T6
Q5
S10
T11
R8
K4
G12
G13
B
MOZ DPF – H3K9ac
Q5
K9ac
T6
T3
A7
K4
T11
R8
G12
C
MOZ DPF – H3K14ac
G12
K9
G13
A1
T11
A7
R8
T3
R2
K4
K14ac

FIGURE S4

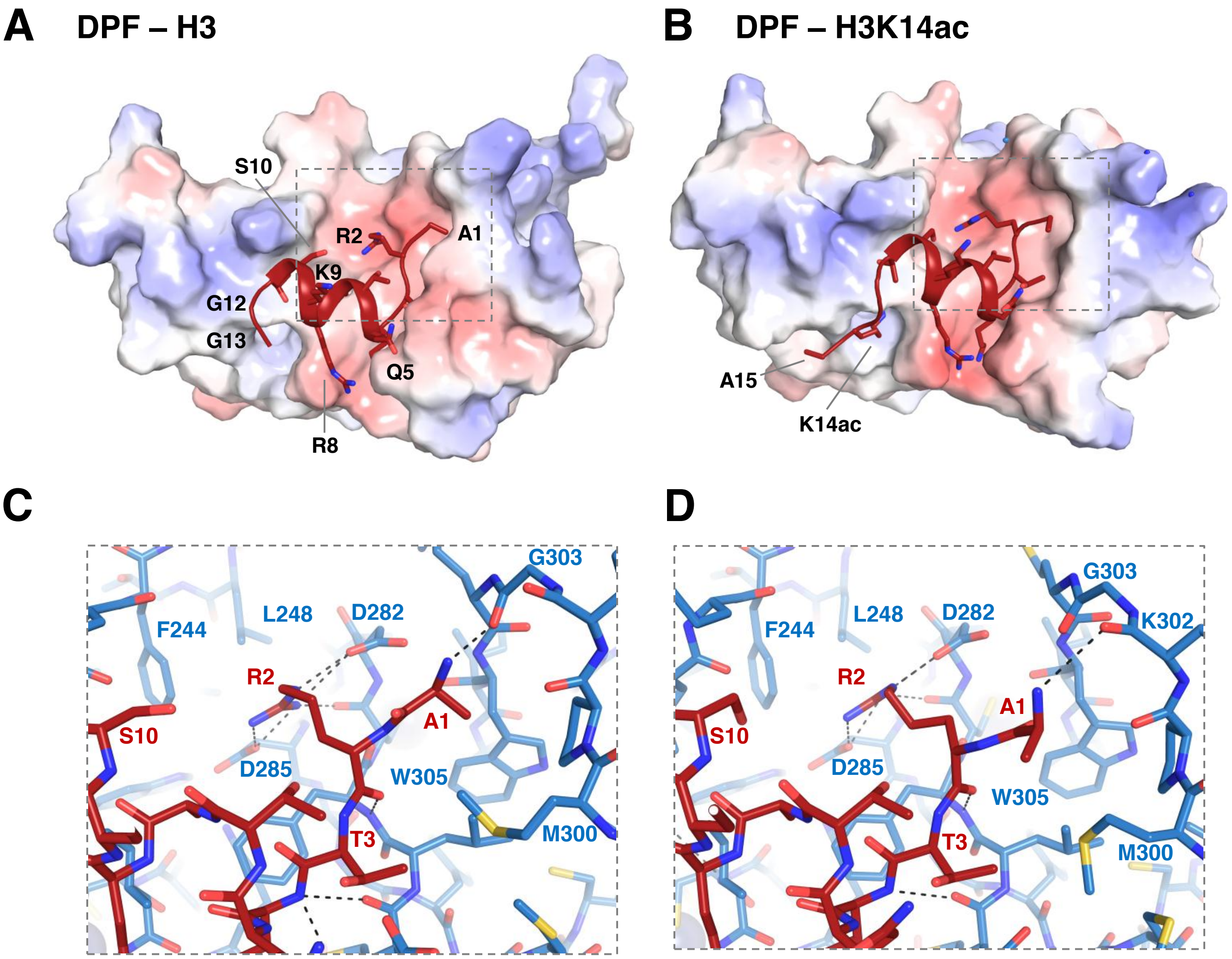
A
DPF – H3
S10
R2
A1
K9
G12
G13
Q5
R8
B
DPF – H3K14ac
A15
K14ac
C
G303
F244
L248
D282
R2
A1
S10
D285
W305
T3
M300
D
G303
K302
F244
L248
D282
R2
A1
S10
D285
W305
T3
M300

FIGURE S5

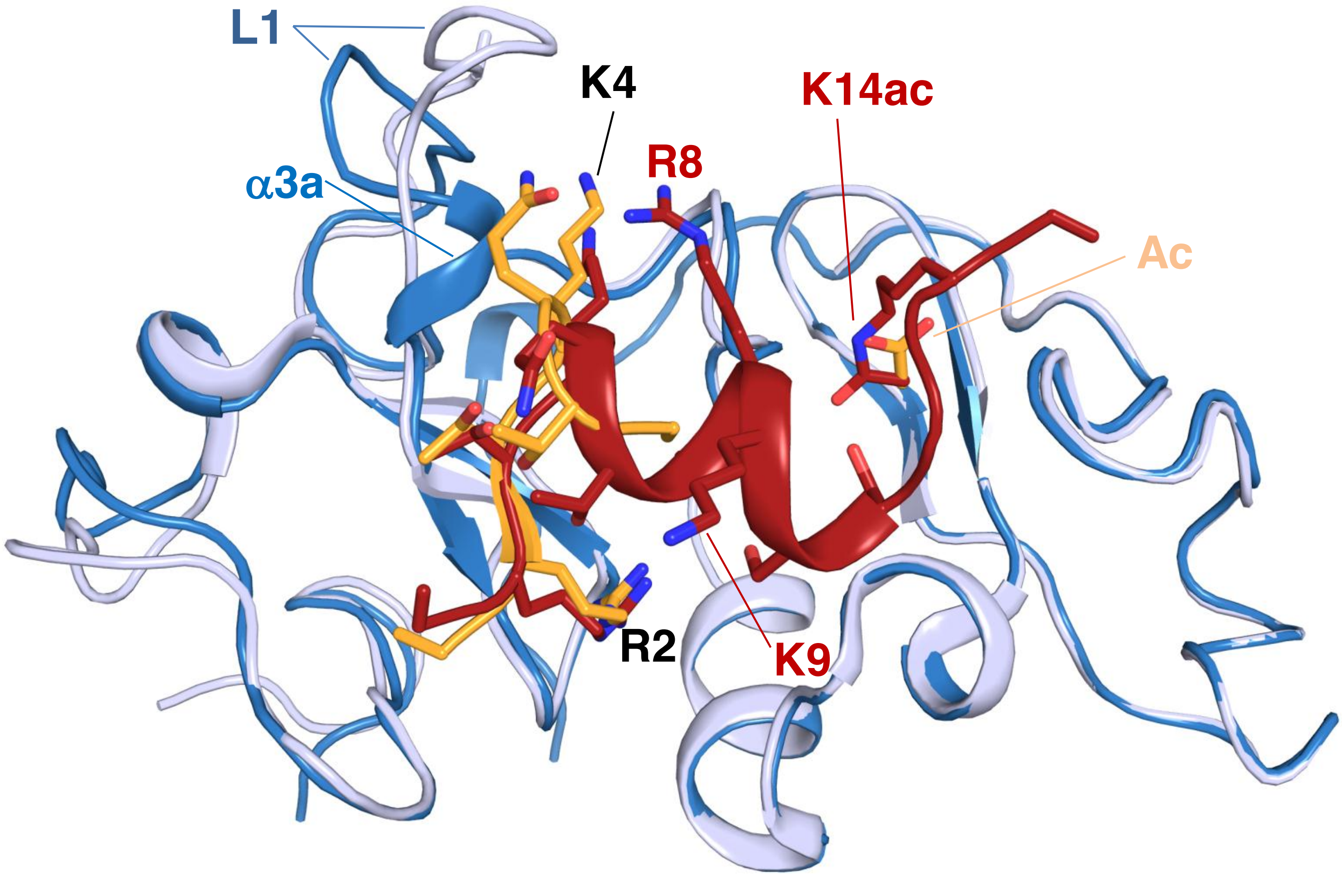

Supplement: Supplementary Data [file supp_gkt931_nar-02188-m-2013-File008.pdf]
